# Statistical indirect associations of sleep disturbance and GAD symptoms in the relationship between childhood trauma and non-suicidal self-injury in adolescents with depression

**DOI:** 10.3389/fpsyg.2026.1823137

**Published:** 2026-06-17

**Authors:** Heng Shao, Ziqiu Xie, Yalu Wu, Tingting Yang, Mengzhu Zhang, Kongliang He

**Affiliations:** 1 School of Mental Health and Psychological Sciences, Anhui Medical University, Hefei, China; 2 Affiliated Psychological Hospital of Anhui Medical University, Hefei Fourth People’s Hospital, Hefei, China

**Keywords:** adolescents, childhood trauma, GAD symptoms, non-suicidal self-injury, sleep disturbance

## Abstract

**Background:**

Childhood trauma is an early stressor for adolescent depression. Previous studies have reported a link between childhood trauma and non-suicidal self-injury (NSSI) behaviors; however, the underlying associations among these variables remain unclear.

**Method:**

Using the Chinese Version of the Functional Assessment of Self-Mutilation, Childhood Trauma Questionnaire Short Form, Pittsburgh Sleep Quality Index, and Generalized Anxiety Disorder-7 scale (GAD-7) as research instruments, we evaluated adolescents with depression who met the ICD-10 criteria for depressive episodes (n = 2,308) from the outpatient clinic of 12 psychiatric hospitals in China.

**Results:**

The scores of sleep disturbance, GAD symptoms, childhood trauma, and their subtypes for the adolescents with depression in the NSSI group were higher than those in the non-NSSI group. Pearson correlation analysis revealed that childhood trauma and its subtypes showed significant positive correlations with NSSI severity, sleep disturbance, and GAD symptoms. PROCESS-based mediation analysis and SEM-based sensitivity analyses showed significant statistical indirect associations between childhood trauma and NSSI behavior through sleep disturbance, GAD symptoms, and the sequential pathway involving sleep disturbance and GAD symptoms. After excluding general confounding factors, emotional abuse, physical abuse, sexual abuse, and physical neglect were significantly associated with NSSI severity in the cross-sectional regression model.

**Conclusion:**

It is essential to remain alert to the increased risk of NSSI behavior among adolescents with depression, particularly those who have experienced different types of childhood trauma. Special consideration should be given to the impact of clinical symptoms such as sleep disturbance and GAD symptoms, and early intervention should be implemented to mitigate or reduce the possibility of NSSI behavior.

## 1 Introduction

Depression is a highly prevalent mental health problem affecting adolescents worldwide, characterized by early onset, high relapse rate, and significant risk of self-harm and suicide (Baldessarini et al., 2017). Globally, around 350 million individuals suffer from depression, with 4–5% of adolescents affected. Their lifetime recurrence rate for depression is alarmingly high, reaching up to 70% (Goldman, 2012). Compared with adults, adolescents with depression may present with unique clinical features, such as somatic complaints, eating disturbances, school refusal, and decline in academic performance, which may increase the likelihood of underdiagnosis (Rice et al., 2019). Adolescents experiencing depression encounter not only the burden posed by the illness itself, but also external pressure from family, school, and peers. These factors can intensify negative emotions, physical discomfort, and increase the risk of engaging in harmful behaviors such as smoking, substance abuse, self-harm, and suicidal tendencies (Wang C. et al., 2024).

A major concern is non-suicidal self-injury (NSSI), a significant risk factor for suicide (Peng et al., 2024; Buelens et al., 2023). Although NSSI is defined as self-injurious behavior without suicidal intent, it is clinically important in adolescents with depression because it may increase the risk of subsequent suicidal behavior. Adolescents with depression who engage in NSSI often experience more severe depressive symptoms, hopelessness, emotion dysregulation, and impulsivity, all of which may heighten suicide risk. Moreover, repeated exposure to self-inflicted pain and injury may increase habituation to pain and reduce fear of self-harm, thereby lowering the threshold for more lethal self-injurious behaviors. Therefore, NSSI may serve not only as a maladaptive strategy for regulating negative affect but also as an important warning sign for potential suicidal behavior in adolescents with depression. Adolescents with depression and NSSI face worse prognosis, longer treatment cycles, and higher suicide risks (Zinchuk et al., 2022). Adverse parent–child relationships may represent an important family-related context for childhood trauma (Gao et al., 2024). When children are exposed to prolonged periods in an unsafe environment, such as emotional neglect, emotional abuse, insufficient parental support, invalidation, or persistent family conflict, they may seek a sense of security through maladaptive coping mechanisms such as resistance, avoidance, or self-harm. These experiences were conceptualized as forms of childhood trauma or adverse childhood experiences rather than merely as contributors to trauma. Childhood trauma may impair adolescents’ sense of security and emotion regulation ability, thereby increasing vulnerability to depressive symptoms and maladaptive coping behaviors. In adolescents with depression, trauma-related emotion dysregulation may increase the likelihood of using NSSI as a maladaptive strategy to temporarily relieve psychological distress or regain a sense of control (Liu et al., 2024). Childhood trauma may serve as a distal etiological risk factor for adolescent depression and may increase vulnerability to depressive symptoms through chronic stress exposure, reduced sense of security, impaired emotion regulation, and maladaptive cognitive patterns (Schmaal and Bendall, 2018). Although several psychosocial interventions have been explored for adolescents who engage in self-harm or NSSI, the current evidence base remains limited. Among these interventions, dialectical behavior therapy for adolescents (DBT-A) has shown relatively consistent evidence in reducing repeated self-harm and related suicidal outcomes. Nevertheless, many adolescents with depression and NSSI remain untreated due to adolescent-specific clinical features of depression, the hidden nature of NSSI, and reluctance to seek help (Witt et al., 2021; McCauley et al., 2018; Kothgassner et al., 2021).

Childhood trauma encompasses abuse, neglect, or violence before age 16, overwhelms a child’s coping capacity, leading to lasting emotional and cognitive impairments (Zavaschi et al., 2002; Edinger et al., 2020). A meta-analysis in China showed 23.1% physical abuse/neglect, 26.0% emotional abuse/neglect, and 8.7% sexual abuse. These experiences heightened risks for depression, low self-esteem, and NSSI (Fang et al., 2015), with sexual abuse being a strong predictor for NSSI (Sucich et al., 2023; Peleikis et al., 2004). Neurobiological studies (Wang et al., 2023; Salokangas et al., 2021; Kim et al., 2018) revealed that trauma dysregulates the prefrontal cortex-amygdala circuit, exacerbating emotional reactivity, sleep disturbances, and information processing in social activities, leading to problems with behavioral regulation.

Early studies have found that the association between anxiety symptom levels and NSSI behavior remains closely related after controlling for factors such as child abuse and depression (Heim and Nemeroff, 2001; Zhang K. et al., 2023). Rodav et al. (2014) found that individuals in the NSSI behavior group showed significantly higher levels of anxiety symptoms and impulsivity than those in the non-NSSI behavior group. Another meta-analysis by Bentley et al. also suggested that people with anxiety symptoms were at higher risk of NSSI behaviors than those without mood disorders (Bentley et al., 2015). These are in line with Nock’s four-factor theoretical model (Nock, 2009), which conceptualizes NSSI as a maladaptive coping strategy, maintained by four distinct reinforcement processes to address their emotional regulation (Agorastos and Olff, 2020; Luo et al., 2022). It has also been shown that the stress/fear response caused by childhood trauma can cause hyperalgesia in brain regions such as the prefrontal cortex, amygdala, and hippocampus, leading to an overreaction of the hypothalamic pituitary adrenal (HPA) axis and sympathetic nervous system, which can contribute to difficulties in the initiation and maintenance of sleep (Pynoos et al., 1999).

It has been suggested that there is a correlation between poor sleep quality and NSSI behavior (Khazaie et al., 2021). First, a longitudinally designed study on sleep involving 392 adolescents showed that sleep quality problems in the 12- to 14-year-old group were effective in predicting the risk of NSSI behavior after 2 to 3 years (Wong et al., 2011). Another study of sleep quality involving 881 adolescents showed that poor sleep only predicted the likelihood of NSSI behavior in females, but could not correctly predict the outcome of male adolescents (Lundh et al., 2012). A cross-sectional study of 15,431 adolescents in China in 2021 (Tang et al., 2021) showed that 68.5% of adolescents slept less than 8 h at night on weekdays, and the prevalence of NSSI behavior within 12 months was 29.4%. Compared with adolescents who slept for 0 ~ 1 h on weekends, adolescents with a make-up sleep time of < 0 h had a higher risk of NSSI behavior. Therefore, it is reasonable to speculate that sleep problems may mediate childhood trauma and NSSI behavior, which requires further research (Zheng et al., 2023).

Although increasingly more studies have focused on NSSI behavior in adolescents with depression, the influencing factors still need to be studied. First, it has been shown that childhood trauma increases the risk of suicide and self-injury through perceived stress and sleep deprivation, especially in adolescents with depression and frequent insomnia, increasing the risk of NSSI behavior (Taylor et al., 2018). Anxiety symptoms are highly comorbid with depression in adolescents and may share common etiological mechanisms, including childhood trauma, dysregulation of the hypothalamic–pituitary–adrenal (HPA) axis, and impaired emotion regulation (Pynoos et al., 1999; Bernstein et al., 2003; Wang Z. et al., 2024). In adolescents with depression, anxiety symptoms may further amplify negative cognitive biases, physiological arousal, and avoidance behaviors, thereby increasing psychological distress and the risk of maladaptive coping strategies such as non-suicidal self-injury (NSSI) (Rodav et al., 2014; Bentley et al., 2015).

Moreover, sleep disturbance and anxiety symptoms exhibit a bidirectional relationship. Sleep disturbance may impair emotion regulation, increase physiological hyperarousal, and intensify negative emotional responses, exacerbating anxiety symptoms; conversely, anxiety symptoms may prolong sleep latency and increase nocturnal arousal, further reducing sleep quality (Khazaie et al., 2021; Wong et al., 2011; Tang et al., 2021). Therefore, in the present study, sleep disturbance was modeled as preceding GAD symptoms based on prior theoretical and empirical considerations to examine a theoretically informed statistical indirect association between childhood trauma and NSSI in adolescents with depression. Therefore, sleep disturbance and GAD symptoms were examined as theoretically informed statistical mediators in the association between childhood trauma and NSSI behavior. Based on theoretical analysis and previous empirical studies, a chain mediation model was established to test the following hypotheses:

*Hypothesis 1:* There is an association between childhood trauma, sleep disturbance, GAD symptoms, and NSSI behavior.

*Hypothesis 2:* Sleep disturbance is involved in a statistical indirect association between childhood trauma and NSSI behavior.

*Hypothesis 3:* GAD symptoms are involved in a statistical indirect association between childhood trauma and NSSI behavior.

*Hypothesis 4:* Sleep disturbance and GAD symptoms are involved in a sequential statistical indirect association between childhood trauma and NSSI behavior.

*Hypothesis 5:* Different subtypes of childhood trauma are differentially associated with NSSI behavior.

## 2 Methods

### 2.1 Participants

From January 2021 to December 2021, a cross-sectional survey was conducted on adolescents with depression at the outpatient clinics of 12 psychiatric hospitals in China. Prior to data collection, the research obtained ethics approval from the Ethics Committee of the Fourth People’s Hospital of Hefei(No: IRB-HFSY-YJ-KYXM-HKL). Participants were identified from the database and approached via simple random sampling. All participants and their guardians were asked to sign written consent before enrollment. They were then filling in questionnaires designed by the research team. A total of 2,411 questionnaires were distributed, and 2,308 valid questionnaires were retrieved, representing 490 males and 1818 females. Among them, 1752 have NSSI behaviors.

#### 2.1.1 Inclusion and exclusion criteria

**Inclusion criteria:**

Meet the ICD-10 diagnostic criteria for a current depressive episode.
The age ranges between 8 and 18 years old.
Those who have received ≥ 6 years of education and can complete the questionnaire independently.
There is no history of suicidal behavior in the past.

**Exclusion criteria:**

Patients with severe drug abuse and organic diseases;
Patients with depressive symptoms caused by other medical conditions;
Patients with severe mental disorders, such as schizophrenia, intellectual disability, bipolar disorder, a history of manic/hypomanic episodes, or other severe mental disorders.

ICD-10 was used because data collection was conducted from January 2021 to December 2021, during which ICD-10 remained the standard diagnostic system used in the participating hospitals and clinical records. Although ICD-11 had been adopted by the World Health Assembly in 2019, it formally came into effect for Member States on January 1, 2022.

### 2.2 Research instrument

The study comprised two parts. The first part focused on the participants’ demographic information. The second part included four validated questionnaires to assess several parameters: the Chinese Version of the Functional Assessment of Self-Mutilation, the Childhood Trauma Questionnaire Short Form, the Pittsburgh Sleep Quality Index, and the Generalized Anxiety Disorder-7 scale.

#### 2.2.1 General information

The general demographic information of the participants, including their age, gender, place of residence, education level, and annual household income, was collected using a self-made questionnaire designed by the research team.

#### 2.2.2 Chinese version of the functional assessment of self-mutilation (C-FASM)

Lloyd-Richardson et al. (2007) developed the Functional Assessment of Self-Mutilation (FASM) to assess self-injurious behavior in individuals. In the present study, the Chinese Version of the Functional Assessment of Self-Mutilation (C-FASM) (Qu et al., 2021) was used to assess NSSI behavior. The C-FASM records whether participants engaged in NSSI behaviors during the past 12 months across 10 different self-injury methods, including cutting or scratching the skin, hitting oneself, biting oneself, tearing one’s hair, and other forms of self-injury. A “yes” response to any of the 10 NSSI methods was considered a positive screen for NSSI behavior. In addition, NSSI severity was assessed using 15 items rated on a 0–3 scale (0 = “never,” 1 = “occasionally,” 2 = “sometimes,” 3 = “often”), with higher total scores indicating more severe NSSI behavior. In the present study, the Cronbach’s *α* of the C-FASM was 0.991.

#### 2.2.3 Childhood Trauma Questionnaire (CTQ) Short Form

The Childhood Trauma Questionnaire Short Form (CTQ-SF) is a 28-item self-report instrument developed by Bernstein et al. to assess childhood trauma, including emotional abuse, physical abuse, sexual abuse, emotional neglect, and physical neglect (Bernstein et al., 2003). The Chinese version of the CTQ-SF has demonstrated acceptable reliability and validity in Chinese populations and has been widely used to assess childhood trauma (Zhao et al., 2005; He et al., 2019). The presence of childhood trauma was determined according to the rating manual used to define the threshold for severe trauma in any subscale (i.e., physical abuse ≥ 10 points, emotional abuse ≥ 13 points, emotional neglect ≥ 15 points, physical neglect ≥ 10 points, sexual abuse ≥ 8 points). A total CTQ score greater than 36 points indicates the presence of childhood trauma, with higher scores indicating more severe childhood trauma. The Cronbach’s *α* for each subtype of CTQ was 0.829 (physical abuse), 0.829 (emotional neglect), 0.740 (emotional abuse), 0.863 (sexual abuse), and 0.504 (physical neglect), respectively.

#### 2.2.4 Pittsburgh Sleep Quality Index (PSQI)

The Pittsburgh Sleep Quality Index (PSQI) was developed by Buysse et al. (1989) to evaluate sleep quality during the past month. The Chinese version of the PSQI has been validated and shown good reliability and validity in Chinese populations (Liu et al., 1996). The PSQI has also been used to assess self-reported sleep quality in clinical populations (Kayrak et al., 2013). It consists of 18 items assessing seven factors: subjective sleep quality, sleep latency, sleep persistence, habitual sleep efficiency, sleep disturbances, use of sleep medications, and daytime dysfunction. Each item was scored on a scale ranging from 0 to 3, and the cumulative score of each component produced the total PSQI score. Total scores ranged from 0 to 21, with higher scores indicating worse sleep quality. Cronbach’s *α* was 0.88.

#### 2.2.5 Generalized Anxiety Disorder-7 scale (GAD-7)

The Generalized Anxiety Disorder-7 scale (GAD-7) was originally developed by Spitzer et al. to assess generalized anxiety symptoms (Spitzer et al., 2006). The Chinese version of the GAD-7 has demonstrated good reliability and validity in Chinese clinical populations (He et al., 2010). In addition, its psychometric properties have also been supported in a large sample of Chinese adolescents (Sun et al., 2021). Therefore, the GAD-7 was used in the present study to assess GAD symptoms during the past two weeks. The GAD-7 total score ranges from 0 to 21. A score of 0–4 is considered minimal GAD symptoms, 5–9 mild, 10–14 moderate, and 15–21 severe. The GAD-7 scale has good psychometric properties, and in this study, Cronbach’s α was 0.920.

### 2.3 Statistical analysis

In this study, SPSS version 25.0 software was used for statistical analysis. Continuous variables were expressed as mean ± standard deviation (M ± SD), and n (%) for categorical variables. Pearson correlation analysis was performed to explore the correlation between childhood trauma, sleep disturbance, GAD symptoms, and NSSI behavior in adolescent depression patients. Model 6 in the PROCESS 4.1 macro was used to examine theoretically informed statistical indirect associations involving sleep disturbance and GAD symptoms between childhood trauma and NSSI behavior. First, the variables were standardized, then the covariates, such as gender, age, and height were controlled. Then model 6 in the PROCESS 4.1 macro program was used to estimate indirect association coefficients and their 95% confidence intervals of sleep disturbance and GAD symptoms between childhood trauma and NSSI behavior. The 95% confidence interval of each path coefficient was calculated by using the deviation correction percentile Bootstrap method, and the 95% confidence interval excluding 0 was used as the statistically significant standard. Finally, hierarchical regression analyses were conducted to examine whether different subtypes of childhood trauma were associated with NSSI severity after adjusting for general covariates, sleep disturbance, and GAD symptoms. Effect sizes were reported in addition to *p* values. For group comparisons, Cohen’s d was calculated for continuous variables, and Cramer’s V was calculated for categorical variables. Pearson’s correlation coefficient r was used as the effect size for correlation analyses. For regression analyses, standardized *β* coefficients and R^2^ values were reported. For mediation analyses, indirect effect estimates, 95% confidence intervals, and effect proportions were reported to quantify the magnitude of the statistical indirect associations. As a sensitivity analysis, observed-variable structural equation modeling was conducted using the lavaan package in R to examine whether the statistical indirect associations were robust to model specification. Three SEM models were estimated: the hypothesized sequential model, in which childhood trauma was associated with NSSI through sleep disturbance and GAD symptoms; an alternative sequential model, in which GAD symptoms preceded sleep disturbance; and a parallel-mediator model, in which sleep disturbance and GAD symptoms were specified as correlated mediators. The same covariates as those in the primary PROCESS analysis, including age, gender, and height, were included. Indirect effects were estimated using 5,000 bootstrap samples with 95% confidence intervals.

## 3 Results

### 3.1 Analysis of differences between the patients with no NSSI and NSSI groups

A total of 2,308 adolescent patients with depression were included in this study, and 1752 patients with NSSI behavior and 556 patients without NSSI behavior were detected, so the detection rate of NSSI behavior was 75.9%. Statistical analysis of general demographic data, CTQ and its subtypes, PSQI, and GAD-7 scores between the two groups is shown in Table 1. There are statistically significant differences in home address and parental education level. The NSSI group had a higher proportion of urban residents and lower parental education levels. A statistically significant difference is observed in the total CTQ scores between the groups with and without NSSI, and the results are significant. The scores for the five CTQ subtypes (emotional abuse, physical abuse, sexual abuse, emotional neglect, and physical neglect) in patients with NSSI behavior were all higher than those in the non-NSSI group, showing significant differences between the two groups, and the results were statistically significant. When comparing the total PSQI scores and GAD-7 scores between the two groups, the NSSI group had higher scores than the non-NSSI group, and the results were statistically significant.

**Table 1:** The differences in variables between the no NSSI and NSSI groups.

Variable	no NSSI (*n* = 556)	NSSI (*n* = 1,752)	*χ*^2^/*t*	*P*	Effect size
Gender (*n* %)					0.003
Male	117 (21.04)	373(21.29)	0.015	0.901	
Female	439(78.96)	1,379(78.71)			
Age (years)	15.04 ± 1.47	15.01 ± 1.66	0.339	0.735	0.019
Home address (*n* %)					0.245
City	219(39.39)	1,181(67.41)	138.859	<0.001	
Countryside	337(60.61)	571(32.59)			
Height (cm)	164.21 ± 7.74	164.10 ± 8.03	0.265	0.791	0.014
Weight (kg)	54.80 ± 11.24	54.81 ± 11.42	−0.018	0.986	0.001
Years of schooling (years)	9.07 ± 1.80	9.20 ± 1.75	−1.507	0.132	0.074
Father’s education level (*n* %)					0.335
No formal education	41(7.37)	215(12.27)	258.432	<0.001	
Elementary school	136(24.46)	904(51.60)			
Junior high school	158(28.42)	419(23.92)			
High school and above	221(39.75)	209(11.93)			
Mother’s education level (*n* %)					0.319
No formal education	59(10.61)	396(22.60)	234.874	<0.001	
Elementary school	135(24.28)	791(45.15)			
Junior high school	153(28.42)	350(19.98)			
High school and above	209(37.59)	215(12.27)			
Childhood trauma	43.31 ± 8.96	51.65 ± 13.30	−16.831	<0.001 ^**^	0.673
Emotional abuse	6.67 ± 2.64	8.27 ± 4.48	−10.308	<0.001 ^**^	0.389
Physical abuse	9.58 ± 2.86	11.16 ± 3.09	−10.681	<0.001 ^**^	0.520
Sexual abuse	12.47 ± 4.97	14.28 ± 4.90	−7.593	<0.001 ^**^	0.368
Emotional neglect	9.58 ± 3.73	11.88 ± 4.94	−11.629	<0.001 ^**^	0.492
Physical neglect	5.01 ± 2.26	6.06 ± 4.11	−7.648	<0.001 ^**^	0.280
PSQI total score	11.67 ± 6.62	12.96 ± 6.20	−4.068	<0.001 ^**^	0.205
GAD-7 total score	9.83 ± 5.61	11.47 ± 5.49	−6.101	<0.001 ^**^	0.297

*p < 0.05, **p < 0.001. Effect sizes are reported as Cohen’s d for continuous variables and Cramer’s V for categorical variables.

### 3.2 An analysis of the patterns and frequency of NSSI behavior

The results are shown in Table 2. Among them, intentionally cutting or scratching one’s own skin was the most common NSSI behavior, accounting for about 66.20%. According to the frequency of occurrence, the order was as follows: intentionally beating oneself (42.20%); deliberately scratching one’s own skin (40.60%); hitting or striking the head with a fist (38.04%); deliberately biting oneself (36.66%); deliberately tearing their own hair (30.59%); deliberately using sharp objects to engrave words and patterns (26.46%); intentional scratching and scratching of oneself caused bleeding (24.05%); deliberately piercing the skin or fingernails with an object (14.43%); intentional irritation of the wound to prevent healing (3.34%).

**Table 2:** Modes and frequency of self-injury.

NSSI behavior	Percentage (%)	Frequency, M ± SD
1. Deliberately cutting or scratching the skin	66.20	62.183 ± 5.03
2. Deliberately assaulting yourself	42.20	31.06 ± 123.36
3. Deliberately tearing your own hair	30.59	24.65 ± 162.06
4. Deliberately using sharp objects to engrave words or patterns	26.46	8.39 ± 45.43
5. Deliberately irritate the wound to prevent healing	3.34	17.24 ± 86.20
6. Deliberately piercing the skin or fingernails with an object	14.43	2.48 ± 14.43
7. Deliberately biting yourself	36.66	8.14 ± 62.70
8. Deliberately scratching or scratching oneself to cause bleeding	24.05	30.90 ± 198.80
9. Hit or hit the head with a fist	38.04	7.32 ± 34.65
10. Deliberately scratching your own skin (knife, pen, etc.)	40.60	4.22 ± 38.03

### 3.3 Correlation analysis of childhood trauma, sleep disturbances, GAD symptoms, and NSSI behavior in adolescents with depression

Table 3 shows the Pearson correlation analysis of childhood trauma and the five subtypes, as well as sleep disturbance, GAD symptoms, and NSSI behavior in adolescent patients with depression. The results showed that the total CTQ score and emotional abuse, physical abuse, sexual abuse, emotional neglect, and physical neglect were significantly positively correlated with the severity of NSSI behavior (*p* < 0.01). The total CTQ score and its five subtypes were significantly positively correlated with the severity of sleep disturbances and the severity of GAD symptoms. There was a significant positive correlation between the severity of sleep disturbance, the severity of GAD symptoms, and the severity of NSSI behavior. There was a significant positive correlation between the severity of GAD symptoms and the severity of NSSI behavior.

**Table 3:** Correlation analysis of childhood trauma and its subtypes, sleep disturbance, GAD symptoms, and NSSI behavior.

Variable	1	2	3	4	5	6	7	8	9
1	1								
2	0.764^**^	1							
3	0.741^**^	0.388^**^	1						
4	0.377^**^	0.262^**^	0.371^**^	1					
5	0.597^**^	0.595^**^	0.239^**^	0.103^**^	1				
6	0.750^**^	0.584^**^	0.474^**^	0.299^**^	0.337^**^	1			
7	0.271^**^	0.172^**^	0.223^**^	0.142^**^	0.130^**^	0.236^**^	1		
8	0.313^**^	0.204^**^	0.259^**^	0.154^**^	0.153^**^	0.252^**^	0.706^**^	1	
9	0.487^**^	0.362^**^	0.376^**^	0.247^**^	0.235^**^	0.446^**^	0.487^**^	0.599^**^	1

*P < 0.05, **P < 0.001. 1 = Total CTQ score, 2 = Emotional abuse score, 3 = Physical abuse score, 4 = Sexual abuse score, 5 = Emotional neglect score, 6 = Physical neglect score, 7 = PSQI score, 8 = GAD-7 score, 9 = NSSI behavior severity score. Pearson’s r values are presented as effect sizes.

### 3.4 Statistical indirect associations of sleep disturbance and GAD symptoms between childhood trauma and NSSI behavior

**Figure 1:**
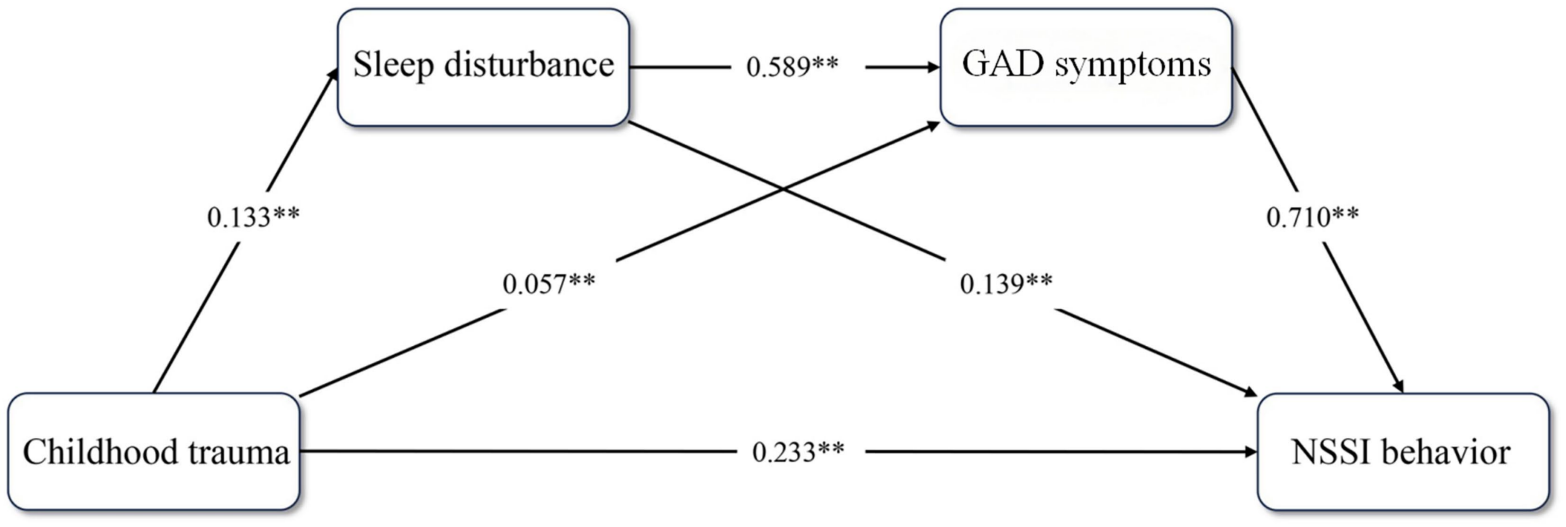
Mediating effects of sleep disturbance and GAD symptoms on the association between childhood trauma and NSSI behaviors in adolescents with depression.

The regression equations for the statistical indirect association model are shown in Table 4. The mediation analysis identified three significant statistical indirect pathways between childhood trauma and NSSI behavior: (1) childhood trauma → sleep disturbance → NSSI behavior, with an effect size of 0.018 and an effect proportion of 5.30%; (2) childhood trauma → GAD symptoms → NSSI behavior, with an effect size of 0.040 and an effect proportion of 11.63%; and (3) childhood trauma → sleep disturbance → GAD symptoms → NSSI behavior, with an effect size of 0.056 and an effect proportion of 16.00% (Table 5; Figure 1).

**Table 4:** Statistical indirect associations among childhood trauma, sleep disturbance, GAD symptoms, and NSSI behavior.

Regression equations (*N* = 2,308)		Fitting the index			Coefficient significance	
Outcome variables	Predictive variables	*R*	*R^2^*	*F*	*β*	*t*
NSSI behavior		0.487	0.237	717.621		
	Childhood trauma				0.347^**^	26.788
Sleep disturbances		0.271	0.073	182.599		
	Childhood trauma				0.133^**^	13.513
GAD symptoms		0.708	0.501	1155.676		
	Childhood trauma				0.057^**^	3.558
	Sleep disturbances				0.589^**^	44.414
NSSI behavior		0.681	0.463	662.485		
	Childhood trauma				0.233^**^	20.267
	Sleep disturbances				0.139^**^	4.416
	GAD symptoms				0.710^**^	19.620

^*^P < 0.05, **P < 0.001 Standardized β coefficients and R^2^ values are presented as effect size indicators.

**Table 5:** Childhood trauma and NSSI behavior: the chain mediating role of sleep disturbances and GAD symptoms.

Effect pathway	Effect size	Effect percentage	SE	95% CI	
				95% CI lower limit	95% CI upper limit
Total effect	0.347		0.013	0.322	0.373
Direct effects					
X → Y	0.233	67.06%	0.011	0.210	0.255
Total indirect effects	0.114	32.94%	0.006	0.102	0.127
Indirect effects					
X → M1 → Y	0.018	5.30%	0.004	0.010	0.027
X → M2 → Y	0.040	11.63%	0.004	0.032	0.049
X → M1 → M2 → Y	0.056	16.00%	0.005	0.047	0.065

X = childhood trauma; M1 = sleep disturbance. M2 = GAD symptoms; Y = NSSI behavior. Indirect effect estimates and effect proportions are reported to quantify the magnitude of statistical indirect associations. Bootstrap CIs are based on 5,000 samples.

### 3.5 SEM-based sensitivity analysis

To further address the concern regarding the use of cross-sectional data in mediation analysis, SEM-based sensitivity analyses were conducted. The hypothesized sequential SEM model showed a significant indirect association through sleep disturbance and GAD symptoms between childhood trauma and NSSI behavior, with an indirect effect of 0.056 and a 95% CI of 0.047–0.065 (Table 6).

**Table 6:** Hierarchical regression model for the subtypes of childhood trauma.

Variable	Model one: *ΔR^2^ = 0.009*		Model two: *ΔR^2^ = 0.359*		Model three:ΔR^2^ = 0.107	
	*β*	*P*	Regression coefficients	*P*	Regression coefficients	*P*
Age	−0.021	0.316	0.003	0.852	0.004	0.771
Gender	0.026	0.211	0.004	0.799	0.016	0.292
Home address	−0.063^*^	0.003	−0.029	0.094	−0.008	0.631
Father’s education	0.024	0.340	0.004	0.838	−0.016	0.384
Mother’s education	−0.088^**^	0.000	−0.026	0.191	−0.007	0.699
Sleep disturbances			0.126^**^	0.000	0.087^**^	0.000
GAD symptoms			0.508^**^	0.000	0.434^**^	0.000
Emotional abuse					0.085^**^	0.000
Physical abuse					0.095^**^	0.000
Sexual abuse					0.048^**^	0.004
Emotional neglect					0.010	0.596
Physical neglect					0.204^**^	0.000

*P < 0.05, **p < 0.01.

The alternative sequential model, in which GAD symptoms preceded sleep disturbance, also showed a significant sequential indirect association, with an indirect effect of 0.015 and a 95% CI of 0.008–0.021. In the parallel-mediator model, both sleep disturbance and GAD symptoms were significantly involved in the statistical indirect associations between childhood trauma and NSSI behavior. The indirect effect through sleep disturbance was 0.018, with a 95% CI of 0.010–0.027, and the indirect effect through GAD symptoms was 0.096, with a 95% CI of 0.084–0.110.

These SEM sensitivity analyses supported the robustness of the statistical indirect associations involving sleep disturbance and GAD symptoms. However, because the alternative mediator-order model was also statistically plausible, the cross-sectional data could not determine the temporal ordering between sleep disturbance and GAD symptoms (Table 7).

**Table 7:** SEM-based sensitivity analyses of statistical indirect associations involving sleep disturbance and GAD symptoms.

Model	Indirect pathway	Effect	95% CI	Interpretation
Hypothesized sequential model	CTQ → PSQI → GAD-7 → NSSI	0.056	0.047–0.065	Significant
Alternative sequential model	CTQ → GAD-7 → PSQI → NSSI	0.015	0.008–0.021	Significant
Parallel-mediator model	CTQ → PSQI → NSSI	0.018	0.010–0.027	Significant
Parallel-mediator model	CTQ → GAD-7 → NSSI	0.096	0.084–0.110	Significant
Parallel-mediator model	Total indirect effect	0.115	0.102–0.127	Significant

CTQ, Childhood Trauma Questionnaire; PSQI, Pittsburgh Sleep Quality Index; GAD-7, Generalized Anxiety Disorder-7 scale; NSSI, non-suicidal self-injury. All models adjusted for age, gender, and height. Indirect effects were estimated using 5,000 bootstrap samples.

### 3.6 Hierarchical regression model

The hierarchical regression model showed that when sleep disturbance, GAD symptoms, and other covariates were controlled, the four subtypes of emotional abuse (*β* = 0.085, *p* < 0.01), physical abuse (*β* = 0.095, *p* < 0.01), sexual abuse (*β* = 0.048, *p* < 0.01), and physical neglect (*β* = 0.204, *p* < 0.01) were significantly associated with NSSI severity among adolescents with depression in the cross-sectional regression model.

## 4 Discussion

This study examined the associations among childhood trauma, sleep disturbance, GAD symptoms, and NSSI behavior in a large clinical sample of adolescents with depression. The main findings were as follows. First, adolescents with NSSI showed higher levels of childhood trauma, sleep disturbance, and GAD symptoms than those without NSSI. Second, childhood trauma and its subtypes were positively associated with NSSI severity, sleep disturbance, and GAD symptoms. Third, PROCESS-based mediation analyses and SEM-based sensitivity analyses showed significant statistical indirect associations between childhood trauma and NSSI behavior through sleep disturbance, GAD symptoms, and the sequential pathway involving sleep disturbance and GAD symptoms. Finally, among the subtypes of childhood trauma, emotional abuse, physical abuse, sexual abuse, and physical neglect were associated with NSSI severity, although findings involving physical neglect should be interpreted cautiously due to its relatively low internal consistency.

The detection rate of NSSI behavior in this study was 75.9%, which should be interpreted with caution. The present sample consisted of adolescents with depression recruited from psychiatric outpatient clinics, representing a high-risk clinical population rather than a community adolescent sample. In addition, the C-FASM assessed NSSI behaviors over the past 12 months and used endorsement of any NSSI method as the criterion for a positive screen. The scale also included relatively mild or easily concealed self-injury behaviors, such as scratching or biting oneself, which may further contribute to a higher detection rate. Therefore, the 75.9% rate should be interpreted as the detection rate of NSSI in this clinical outpatient sample rather than as a general prevalence estimate among adolescents.

Regarding NSSI methods, deliberately cutting or scratching oneself was the most common form of NSSI behavior, accounting for approximately 66.20% of all NSSI behaviors in this sample. This finding is consistent with previous evidence that adolescents may choose easily accessible body parts and relatively concealed self-injury methods to regulate negative affect or relieve psychological distress (Anestis et al., 2015; Case et al., 2020). The high frequency of cutting or scratching also suggests that clinicians should not limit assessment to severe or medically dangerous self-injury, but should also ask about less visible or more easily concealed behaviors.

### 4.1 Sleep disturbance in the association between childhood trauma and NSSI behavior

This study found a significant positive association between childhood trauma and poorer sleep quality among adolescents with depression, which is consistent with previous studies (Montgomery and Foldspang, 2001; Hall Brown et al., 2016). Early adverse experiences may be related to chronic hyperarousal, disrupted sleep regulation, and insomnia symptoms. From the perspective of the 3P model of insomnia, childhood trauma may function as a distal vulnerability factor, while stress-related hyperarousal may contribute to the persistence of sleep difficulties (Perlis et al., 1997; Spielman et al., 1996).

Sleep disturbance may also be clinically relevant to NSSI in adolescents with depression. Previous studies have suggested that sleep problems may be associated with increased impulsivity, impaired emotion regulation, and reduced self-control, all of which are relevant to NSSI behavior (Liu et al., 2017; Tubbs et al., 2022). In adolescents with depression, poor sleep quality may increase vulnerability to negative emotional states and reduce the ability to use adaptive coping strategies. Therefore, sleep disturbance may represent an important clinical marker associated with NSSI severity. However, given the cross-sectional design of the present study, these findings should be interpreted as associations rather than evidence that childhood trauma causes sleep disturbance or that sleep disturbance causes NSSI behavior.

### 4.2 GAD symptoms in the association between childhood trauma and NSSI behavior

This study also found that childhood trauma was positively associated with GAD symptoms, and that GAD symptoms were associated with NSSI severity. These findings are consistent with previous evidence indicating that long-term childhood trauma is related to later anxiety-related symptoms and broader emotional dysregulation (Goodman et al., 2022; King, 2021; Kuzminskaite et al., 2022). In adolescents with depression, childhood trauma may be associated with heightened sensitivity to perceived stress, interpersonal threat, and fear of abandonment. These experiences may contribute to persistent worry, physiological arousal, and difficulty regulating negative affect.

The association between GAD symptoms and NSSI behavior may be understood within emotion regulation models of self-injury. Adolescents with depression who experience intense worry or anxiety-related arousal may use NSSI as a maladaptive strategy to temporarily reduce distress or regain a sense of control. This interpretation is consistent with prior studies suggesting that anxiety-related symptoms and negative emotional responses are associated with self-injurious behaviors (Zhao and Zhou, 2024; Zou et al., 2024; Zhou et al., 2024). Nevertheless, because the present study used cross-sectional data, GAD symptoms should be interpreted as a statistical mediator rather than as a confirmed causal mechanism linking childhood trauma to NSSI.

### 4.3 Statistical indirect associations involving sleep disturbance and GAD symptoms between childhood trauma and NSSI behavior

The results are consistent with Hypothesis 4. Sleep disturbance and GAD symptoms were involved in a significant statistical indirect association between childhood trauma and NSSI behavior in adolescents with depression. Although the hypothesized sequence of sleep disturbance preceding GAD symptoms was based on prior theoretical and empirical considerations, other mediator orderings may also be statistically plausible. For example, GAD symptoms may be associated with poorer sleep quality, and sleep disturbance and GAD symptoms may influence each other bidirectionally. Therefore, the present findings should be interpreted as theoretically informed statistical indirect associations rather than confirmation of a unique temporal pathway.

Previous studies have mainly focused on the role of a single mediator, whereas the present study examined the joint statistical indirect associations of sleep disturbance and GAD symptoms between childhood trauma and NSSI behavior. Childhood trauma, as a distal vulnerability factor for adolescent depression, may be associated with poorer sleep quality and altered functioning of emotion-related brain regions, which may further be related to increased negative emotions such as GAD symptoms. In adolescents with depression, GAD symptoms may intensify psychological distress and increase the likelihood of using NSSI as a maladaptive strategy to regulate negative affect or regain a sense of control.

However, given the cross-sectional design of the present study, this sequential association should be interpreted with caution. The current findings do not establish that sleep disturbance temporally precedes GAD symptoms or that GAD symptoms temporally precede NSSI behavior. Rather, the results suggest that sleep disturbance and GAD symptoms may jointly characterize an important psychological and behavioral risk profile among adolescents with depression who have experienced childhood trauma. This is also consistent with the view that sleep quality may be independently associated with self-injury and self-harm, although it may not necessarily mediate the link between childhood trauma and self-harm attempts in all studies (Kang et al., 2014). More longitudinal studies are needed in the future to clarify the temporal order and potential directional relationships among childhood trauma, sleep disturbance, GAD symptoms, and NSSI behavior.

The SEM-based sensitivity analyses provided additional support for the statistical indirect associations involving sleep disturbance and GAD symptoms. The hypothesized sequential SEM model replicated the main indirect pathway observed in the PROCESS analysis. However, the alternative mediator-order model, in which GAD symptoms preceded sleep disturbance, also yielded a significant indirect association. This finding indicates that although sleep disturbance and GAD symptoms may jointly characterize an important psychological and behavioral risk profile among adolescents with depression who have experienced childhood trauma, the present cross-sectional data cannot distinguish the temporal ordering between these two variables. Therefore, the sequential pathway should be interpreted as a theoretically informed statistical indirect association rather than evidence of a confirmed temporal or causal mechanism.

### 4.4 Childhood trauma subtypes and NSSI behavior

To further examine the associations between different subtypes of childhood trauma and NSSI behavior, this study used hierarchical regression analysis after adjusting for relevant covariates. The findings showed that emotional abuse, physical abuse, sexual abuse, and physical neglect were associated with NSSI severity among adolescents with depression. These findings are broadly consistent with previous studies suggesting that specific forms of childhood trauma, especially emotional abuse and sexual abuse, may be closely related to NSSI behavior (Heleniak et al., 2016; Steine et al., 2012; Zhang L. et al., 2023; Wang H. et al., 2024).

Emotional abuse may be particularly relevant to NSSI because it can undermine adolescents’ self-worth, increase shame and self-criticism, and impair the development of adaptive emotion regulation strategies. Sexual abuse and physical abuse may also be associated with heightened threat sensitivity, traumatic memories, and difficulties in emotional and behavioral regulation. These trauma-related vulnerabilities may increase the likelihood that adolescents with depression use NSSI as a maladaptive coping strategy.

In contrast to some previous findings, emotional neglect was not significantly associated with NSSI severity in the fully adjusted model. This inconsistency may be related to differences in sample characteristics, cultural background, measurement methods, and clinical severity. Moreover, the association between physical neglect and NSSI should be interpreted cautiously because the internal consistency of the physical neglect subscale was relatively low in this study. Therefore, findings involving physical neglect should be regarded as exploratory and require further verification in future studies using more reliable or multi-method assessments. Network analysis has also suggested that, except for emotional abuse, the association between childhood trauma subtype networks and NSSI behavior networks may be relatively weak (Lei et al., 2024), further supporting the need for more detailed subtype-specific research.

### 4.5 Clinical implications

Clinically, these findings suggest that adolescents with depression, especially those with a history of childhood trauma, should be routinely screened for NSSI behavior, sleep disturbance, and GAD symptoms. The high detection rate of NSSI in this outpatient sample indicates that NSSI assessment should not be limited to suicidal intent, but should include a broad evaluation of self-injury methods, frequency, and severity. Less visible behaviors, such as scratching, biting, or hitting oneself, should also be included in routine clinical assessment.

In addition, sleep disturbance and GAD symptoms may jointly indicate a higher-risk psychological profile among adolescents with depression. Early identification of sleep problems and GAD symptoms may help clinicians recognize adolescents who require closer monitoring and more comprehensive intervention. For adolescents with depression and childhood trauma histories, interventions that address sleep problems, anxiety-related symptoms, and maladaptive emotion regulation may be useful components of NSSI prevention and management. Given the cross-sectional nature of the present study, these implications should be understood as clinical screening and risk-identification suggestions rather than evidence of causal intervention targets.

## 5 Limitations and future directions

This study had several limitations. First, because this study used a cross-sectional design, the mediation model cannot establish temporal precedence or causal relationships among childhood trauma, sleep disturbance, GAD symptoms, and NSSI behavior. The proposed sequence of childhood trauma → sleep disturbance → GAD symptoms → NSSI was based on theoretical considerations and prior empirical findings, but the directionality of these associations cannot be confirmed in the present study. In addition, because sleep disturbance and GAD symptoms were measured simultaneously, the ordering of these mediators remains tentative. Alternative models, such as childhood trauma → GAD symptoms → sleep disturbance → NSSI, may also be statistically plausible. Therefore, the mediation findings should be interpreted as statistical indirect associations rather than evidence of causal mechanisms. Although the SEM sensitivity analyses suggested that the indirect associations were robust across different model specifications, SEM based on cross-sectional data cannot resolve temporal ambiguity or eliminate potential bias in indirect effect estimates. Therefore, these findings should not be interpreted as evidence that sleep disturbance temporally precedes GAD symptoms or that either variable causally mediates the association between childhood trauma and NSSI behavior. Future longitudinal or prospective studies are needed to determine whether sleep disturbance precedes GAD symptoms, whether GAD symptoms precede sleep disturbance, or whether the two influence each other bidirectionally over time.

Second, depression severity and medication status were not included as covariates because standardized depression severity scores and detailed medication information were not consistently collected across all participating centers. This may have introduced residual confounding, given the close clinical relationship among depressive symptoms, GAD symptoms, sleep disturbance, and NSSI behavior. In particular, adjustment for depression severity would have helped to better isolate the association between GAD symptoms and NSSI behavior. Future studies should include standardized measures of depression severity and detailed medication information to further test the robustness of these associations.

Third, the Cronbach’s *α* of the physical neglect subscale was relatively low in this sample (α = 0.504), suggesting limited internal consistency. Therefore, findings involving physical neglect should be interpreted cautiously and regarded as exploratory. Future studies should verify these findings using more reliable or multi-method assessments of physical neglect.

Fourth, this study relied on self-report questionnaires, and recall bias may have affected the assessment of childhood trauma and NSSI behaviors. Future studies should combine self-report measures with clinical interviews, multi-informant assessments, or longitudinal follow-up designs to improve the reliability and validity of the findings.

In future studies, there is a need to investigate the impacts of different types of sleep disturbances and childhood trauma on the risk of developing NSSI behaviors. In addition, more attention should be paid to the impact of other adverse emotions (such as anxiety and loneliness) on the behavior pattern and frequency of NSSI, in addition to depressive symptoms, to improve the clinical symptoms involved in adolescent mental illness and reduce the risk of adverse outcomes such as suicide and self-injury.

## Data Availability

The raw data supporting the conclusions of this article will be made available by the authors, without undue reservation.
